# Differences in Use of Clinical Decision Support Tools and Implementation of Aspirin, Blood Pressure Control, Cholesterol Management, and Smoking Cessation Quality Metrics in Small Practices by Race and Sex

**DOI:** 10.1001/jamanetworkopen.2023.26905

**Published:** 2023-08-02

**Authors:** Madeline M. Roberts, Miguel Marino, Rebecca Wells, Folefac D. Atem, Bijal A. Balasubramanian

**Affiliations:** 1 Department of Epidemiology, Human Genetics, and Environmental Sciences, University of Texas Health Science Center at Houston (UTHealth Houston) School of Public Health, Dallas; 2 Department of Family Medicine, Oregon Health & Science University, Portland; 3 School of Public Health, Oregon Health & Science University, Portland; 4 Department of Management, Policy, & Community Health, UTHealth Houston School of Public Health, Houston; 5 Department of Biostatistics and Data Science, UTHealth Houston School of Public Health, Dallas

## Abstract

**Importance:**

Practice-level evidence is needed to clarify the value of population-based clinical decision support (CDS) tools in reducing racial and sex disparities in cardiovascular care.

**Objective:**

To evaluate the association between CDS tools and racial and sex disparities in the aspirin use, blood pressure control, cholesterol management, and smoking cessation (ABCS) care quality metrics among smaller primary care practices.

**Design, Setting, and Participants:**

This cross-sectional study used practice-level data from the Agency for Healthcare Research and Quality–funded EvidenceNOW initiative. The national initiative from May 1, 2015, to April 30, 2021, spanned 12 US states and focused on improving cardiovascular preventive care by providing quality improvement support to smaller primary care practices. A total of 576 primary care practices in EvidenceNOW submitted both survey data and electronic health record (EHR)–derived ABCS data stratified by race and sex.

**Main Outcomes and Measures:**

Practice-level estimates of disparities between Black and White patients and between male and female patients were calculated as the difference in proportions of eligible patients within each practice meeting ABCS care quality metrics. The association between CDS tools (EHR prompts, standing orders, and clinical registries) and disparities was evaluated by multiply imputed multivariable models for each CDS tool, adjusted for practice rurality, ownership, and size.

**Results:**

Across the 576 practices included in the analysis, 219 (38.0%) had patient panels that were more than half White and 327 (56.8%) had panels that were more than half women. The proportion of White compared with Black patients meeting metrics for blood pressure (difference, 5.16% [95% CI, 4.29%-6.02%]; *P* < .001) and cholesterol management (difference, 1.49% [95% CI, 0.04%-2.93%] *P* = .04) was higher; the proportion of men meeting metrics for aspirin use (difference, 4.36% [95% CI, 3.34%-5.38%]; *P* < .001) and cholesterol management (difference, 3.88% [95% CI, 3.14%-4.63%]; *P* < .001) was higher compared with women. Conversely, the proportion of women meeting practice blood pressure control (difference, −1.80% [95% CI, −2.32% to −1.28%]; *P* < .001) and smoking cessation counseling (difference, −1.67% [95% CI, −2.38% to −0.95%]; *P* < .001) metrics was higher compared with men. Use of CDS tools was not associated with differences in race or sex disparities except for the smoking metric. Practices using CDS tools showed a higher proportion of men meeting the smoking counseling metric than women (coefficient, 3.82 [95% CI, 0.95-6.68]; *P* = .009).

**Conclusions and Relevance:**

The findings of this cross-sectional study suggest that practices using CDS tools had small disparities that were not statistically significant, but CDS tools were not associated with reductions in disparities. More research is needed on effective practice-level interventions to mitigate disparities.

## Key Points

**Question:**

Are clinical decision support (CDS) tools associated with lower racial and sex disparities in the aspirin, blood pressure control, cholesterol management, and smoking cessation (ABCS) quality metrics of cardiovascular prevention among smaller primary care practices?

**Findings:**

This cross-sectional study of 576 primary care practices showed significant race and sex disparities in ABCS quality metrics. Practices using CDS tools had small disparity estimates that were not statistically significant.

**Meaning:**

These findings suggest that race and sex disparities exist in cardiovascular prevention quality metrics among smaller primary care practices; effective interventions are needed in this setting.

## Teaser

This cross-sectional study evaluated the association between primary care practice use of electronic health record prompts, standing orders, and clinical registries and disparities by race and sex in practice-level care quality metrics consisting of aspirin use, blood pressure control, cholesterol management, and smoking cessation.

## Introduction

One in 5 US deaths is attributable to cardiovascular disease (CVD), and 49% of adults have some form of CVD; thus, small disparities in prevention and management can substantively affect morbidity and mortality.^1^ Racial and sex disparities in CVD prevention and management are well-documented. Black patients and women are less likely to be prescribed cardioprotective medications compared with White patients and men.^2,3,4,5,6^ Primary care physicians report lower implementation of CVD evidence-based guidelines among women and prioritize weight loss and breast health, despite heart disease being the leading cause of mortality among women.^7,8,9^

The Million Hearts initiative emphasizes 4 evidence-based measures for secondary preventive care and CVD management.^10^ Aspirin use for at-risk individuals aged 40 to 59 years, blood pressure control for hypertension, cholesterol management, and smoking cessation counseling are together referred to as the ABCS of CVD prevention.^10^

Electronic health records (EHR) prompts, standing orders, and clinical registries are clinical decision support (CDS) tools, which typically function as strategies to improve patient outcomes.^11,12,13^ Less is known about their effect on disparities in delivering guideline-concordant care within primary care.^14^ Prompts in the EHR and standing orders are point-of-care tools that guide clinician and care team behavior. Clinical registries help manage chronic conditions by identifying services due and gaps in care or tracking patient progress. Protocol-driven processes are associated with fewer racial disparities in CVD management among hospitals, presumably a result of care standardization and mitigating bias.^15^ Research is needed, however, to clarify whether use of CDS tools reduces disparities in care delivery among smaller independent practices, which provide most primary care nationally.^16^ These clinics often lack resources to implement evidence-based practices.^17,18^

Here, we focus on 3 CDS tools: EHR prompts, standing orders, and clinical registries. Using practice-level data from a multiple-state sample of smaller primary care practices in EvidenceNOW, we aimed to evaluate the association between practice use of these CDS tools and race and sex disparities in practice-level ABCS quality metrics. We hypothesized that because these tools are automated and evidence-based, use of CDS tools in primary care practices would be associated with reductions in race and sex disparities in meeting ABCS quality metrics.

## Methods

This cross-sectional study is part of the Evaluating System Change to Advance Learning and Take Evidence to Scale (ESCALATES) study.^19^ The study was approved by the institutional review boards of Oregon Health & Science University and University of Texas Health Science Center, which waived the need for informed consent owing to the use of practice-level data vs individual patient data. This study followed the Strengthening the Reporting of Observational Studies in Epidemiology (STROBE) reporting guideline. EvidenceNOW was a national initiative funded by the Agency for Healthcare Research and Quality from May 1, 2015, to April 30, 2021, to support smaller primary care practices (<10 physicians per clinic) making quality improvements to enhance the ABCS metrics of cardiovascular preventive care delivery. Seven research teams, which the Agency for Health Care Research and Quality termed *cooperatives*, received grants to provide quality improvement support for ABCS measures among 1720 smaller practices spanning 12 states.^20^

### Study Design and Data Sources

This cross-sectional analysis used baseline data from May 1, 2015, to December 31, 2016 (before any interventions started), collected as part of the external evaluation of EvidenceNOW. Two types of practice-level data are included in this analysis: (1) ABCS quality metrics stratified by race and sex and (2) practice surveys. Patient-level data were outside the scope and access of this initiative.

Data on practice ABCS quality metrics stratified by race and sex were derived from practices’ EHR data, collected by cooperatives, and shared with the evaluation team. The ABCS metrics were defined by Centers for Medicare & Medicaid Services (eTable 2 in Supplement 1). We restricted the sample to practices where concordant pairs existed, that is, both Black and White patients, and enough in each category to meet minimum denominator criteria for data quality (≥10 patients for aspirin; ≥30 patients each for blood pressure, cholesterol management, and smoking cessation). Race information in practice EHRs is most often derived through patient self-report collected by practices.

Practice surveys, typically completed by practice managers, ascertained data on use of CDS tools and practice characteristics, including demographic characteristics of patient panels (Table 1). Respondents reported percentages of patients in their practice panel, which was to sum to 100%, in the following categories: American Indian or Alaska Native, Asian, Black or African American, Native Hawaiian or Other Pacific Islander, White, some other race, or more than 1 race. They were instructed to obtain this information from practice management systems.

**Table 1.** Characteristics of EvidenceNOW Practices With Both Survey Data and Aspirin Use, Blood Pressure Control, Cholesterol Management, and Smoking Cessation Performance Data Stratified by Race and Sex

Characteristic	No. (%) of practices (N = 576)^a^
Practice	
Location	
Urban	424 (73.6)
Suburban	49 (8.5)
Large town	38 (6.6)
Rural town	65 (11.3)
Size	
1 Clinician	150 (26.0)
2-5 Clinicians	223 (38.7)
6-10 Clinicians	71 (12.3)
≥11 Clinicians	65 (11.3)
Ownership	
Clinician owned	290 (50.3)
Hospital, health system, or HMO	116 (20.1)
FQHC	60 (10.4)
Other^b^	62 (10.8)
Classified as a medically underserved area	
Yes	240 (41.7)
No	257 (44.6)
Received external incentives in prior 12 mo	
Yes	328 (56.9)
No	155 (26.9)
Participation in other demonstration projects	
Yes	163 (28.3)
No	276 (47.9)
ONC-certified EHR	
Yes	437 (75.9)
No	3 (0.5)
Participation in Medicare Promoting Interoperability Program	
Not participating	58 (10.1)
Stage 1 only	76 (13.2)
Stages 1 and 2	302 (52.4)
Produced quality report(s) in prior 6 mo	
Yes	402 (69.8)
No	76 (13.2)
Use of ≥1 registry	
Yes	380 (66.0)
No	97 (16.8)
Guidelines for CVD prevention	
Not used or clinician agreement to use	122 (21.2)
Included in EHR prompts or standing orders	361 (62.7)
Guidelines for CVD management	
Not used or clinician agreement to use	144 (25.0)
Included in EHR prompts or standing orders	339 (58.9)
Patient panel	
White race	
≤50%	209 (36.3)
>50%	219 (38.0)
Missing	148 (25.7)
Women	
≤50%	127 (22.0)
>50%	327 (56.8)
Missing	122 (21.2)

Abbreviations: CVD, cardiovascular disease; EHR, electronic health record; FQHC, federally qualified health center; HMO, health maintenance organization; ONC, Office of the National Coordinator for Health Information.

^a^ Percentages may not total 100% owing to missing data.

^b^ Includes nonfederal government clinics (eg, state, county, city, public health clinic), academic health centers and/or faculty practices, and rural health clinics.

### Practice Inclusion Criteria

Of 1720 EvidenceNOW practices, 783 from 3 cooperatives submitted practice-level race- and sex-stratified ABCS quality metric data. Of these, 576 also submitted survey data. These 576 practices, with both stratified ABCS data and survey data, comprised the analytic sample (eFigure in Supplement 1). Four cooperatives did not provide these data because their practices did not have accurate data on ABCS metrics stratified by race.

### Outcomes

The primary outcome was practice-level race and sex disparities in ABCS quality metrics. For each practice, we obtained the proportion of eligible patients receiving ABCS preventive services stratified by race (White and Black) and sex (male and female), and for each ABCS measure, we calculated the practice-level difference in proportion of White and Black patients and male and female patients meeting ABCS quality metrics.

### Independent Variables

The main independent variable was practice use of CDS tools. This included disease registries and use of CVD prevention and management guidelines in EHR prompts and standing orders. We coded practices as using a disease registry if they responded “yes” to using 1 or more registries to identify services due or gaps in care or to track progress (eg, hypertension, high cholesterol levels).

Practices were asked to identify how they used clinical guidelines both for CVD prevention (ie, smoking cessation intervention) and chronic disease management (ie, use of antithrombotics) based on 5 (yes or no) survey items: (1) practice does not follow specific guidelines; (2) guidelines are posted or distributed in the practice; (3) we have clinician agreement to use specific guidelines; (4) practice uses standing orders (protocols authorizing health team members to complete specific tasks for specific conditions [ie, order laboratory evaluations for patients with diabetes]); and (5) practice uses EHR provider guideline–based prompts and reminders (ie, point-of-care prompt to prescribe statin therapy for patients who meet criteria for low-density lipoprotein cholesterol levels).^20^ We created a dichotomous variable by categorizing practices that responded yes to items 4 or 5 as using EHR prompts and standing orders and categorizing practices that responded yes to 1, 2, or 3 as not using them. This approach was conservative in that while posting, distributing, and agreeing to guidelines represent incremental improvements over not following guidelines, they do not denote active use of EHR prompts or standing orders. Only these 3 CDS tools were included in EvidenceNOW.^20^ We did not have information on how practices used these tools.

### Covariates

Practice rurality, size, and ownership were included as covariates. Use of these covariates was guided by patient-level studies showing these factors could also affect disparities in care,^21,22,23,24,25,26,27,28^ as well as associations within the full cohort of EvidenceNOW.^29^

### Statistical Analysis

Counts and percentages were estimated to characterize practices. We performed 1-sample *t* tests to evaluate race and sex disparities in ABCS metrics. Because of their distinct characteristics, federally qualified health centers (FQHCs) were analyzed separately.

For each ABCS measure, we hypothesized that use of CDS tools would be associated with reductions in ABCS race and sex disparities. To test this, we constructed linear regression models where the outcome was the race and sex disparity estimate for each of the 4 ABCS measures (eg, practice-level difference in blood pressure control between Black and White patients at the same practice), and the independent variables included CDS tool use variables. All models controlled for practice rurality, ownership, and size. Missing data were present in our independent variables; thus, we used multiple imputation by chained equations with 10 imputed data sets for each model to address missingness.^30^

We conducted 4 sensitivity analyses. First, we ran χ^2^ tests comparing characteristics of practices within the analytic sample and excluded practices that submitted survey data. Second, we compared disparity estimates among practices within the analytic sample with excluded practices that submitted stratified ABCS data but not survey data. Third, to evaluate an alternative approach to missing data, we reran regression models, this time including a category for missing values. Last, we evaluated associations between the number of CDS tools practices used and disparity estimates by rerunning regression models, coding CDS tools as a single variable with 4 levels (0, 1, 2, or 3 CDS tools).

All statistical tests were 2-sided and were performed using Stata SE, version 15.1 (StataCorp LLC). *P* < .05 indicated statistical significance. As this was not a causal study, no multiple comparisons adjustment was performed.

## Results

Of the 576 study practices, 219 (38.0%) had patient panels that were more than half White, and 327 (56.8%), more than half women (Table 1). Practices were predominately urban (424 [73.6%]), comprised only 1 clinician (150 [26.0%]) or 2 to 5 clinicians (223 [38.7%]), and were clinician-owned (290 [50.3%]); 60 (10.4%) were FQHCs. Practices used the Office of the National Coordinator for Health Information–certified EHRs (437 [75.9%]) and participated in both stages 1 and 2 of the Medicare Promoting Interoperability Program (formerly, the EHR Incentive Program, or Meaningful Use) (302 [52.4%]). Most practices used at least 1 registry (380 [66.0%]) and implemented guidelines for CVD prevention (361 [62.7%]) and CVD management (339 [58.9%]) in EHR prompts or standing orders.

Table 2 and Table 3 show mean practice-level disparities in ABCS quality metrics in the study sample. On average, the blood pressure control metric was higher for White compared with Black patients (difference, 5.16% [95% CI, 4.29%-6.02%]; *P* < .001). This was also the case for the cholesterol management metric (difference, 1.49% [95% CI, 0.04%-2.93%] *P* = .04).

**Table 2.** Sample Practice-Level Health Disparities by Race

Preventive service	Practice peformance, mean (SD), %		Disparity estimate, mean (SD) [95% CI], %	One-sample *t* statistic
	White	Black		
Aspirin use (187 practices)	59.83 (0.30)	60.41 (0.29)	0.58 (0.12) [−2.32 to 1.16]	−0.65
Blood pressure control (240 practices)	68.08 (0.14)	62.92 (0.15)	5.16 (0.07) [4.29 to 6.02]	11.78^a^
Cholesterol management (166 practices)	62.18 (0.20)	60.70 (0.18)	1.49 (0.09) [0.04 to 2.93]	2.02^b^
Smoking cessation (179 practices)	54.32 (0.35)	55.23 (0.36)	−0.91 (0.06) [−1.85 to 0.03]	−1.91

^a^ *P* < .001.

^b^ *P* = .04.

**Table 3.** Sample Practice-Level Health Disparities by Sex

Preventive service	Practice peformance, mean (SD), %		Disparity estimate, mean (SD) [95% CI], %	One-sample *t* statistic
	Men	Women		
Aspirin use (403 practices)	60.34 (0.29)	55.98 (0.28)	4.36 (0.10) [3.34 to 5.38]	8.43^a^
Blood pressure control (450 practices)	65.37 (0.14)	67.17 (0.14)	−1.80 (0.06) [−2.32 to −1.28]	−6.77^a^
Cholesterol management (400 practices)	61.39 (0.01)	57.50 (0.01)	3.88 (0.08) [3.14 to 4.63]	10.25^a^
Smoking cessation (356 practices)	49.48 (0.33)	51.14 (0.33)	−1.67 (0.07) [−2.38 to −0.95]	−4.60^a^

^a^ *P* < .001.

With respect to sex disparities, the metrics for aspirin use (difference, 4.36% [95% CI, 3.34%-5.38%]; *P* < .001) and cholesterol management (difference, 3.88% [95% CI, 3.14%-4.63%]; *P* < .001) were higher for men compared with women. Conversely, a higher proportion of women met blood pressure control (difference, −1.80% [95% CI, −2.32% to −1.28%]; *P* < .001) and smoking cessation counseling (difference, −1.67% [95% CI, −2.38% to −0.95%]; *P* < .001) metrics compared with men.

Stratified analyses for FQHCs (eTables 3 and 4 in Supplement 1) show that FQHCs had moderately fewer disparities compared with non-FQHCs. The FQHCs had the only instance in our study of a higher proportion of Black patients meeting an ABCS metric than White patients (smoking cessation difference, −1.99% [95% CI, −3.69% to −0.30%]; *P* = .02).

Results from multiply imputed multivariable models are reported in Table 4 and Table 5. Compared with practices that did not use CDS tools, practices that did had varying differences in race disparity estimates (sometimes lessening, sometimes worsening), though the effect sizes were small and not statistically significant. Similarly, among men and women, we found no association between use of CDS tools and sex disparities with one exception—men received higher guideline-concordant care for smoking cessation counseling than women when chronic care guidelines were included in EHR prompts and standing orders (coefficient, 3.82 [95% CI, 0.95-6.68]; *P* = .009).

**Table 4.** Multiply Imputed Multivariable Analysis: Association Between CDS Tools and Racial Disparities in Preventive Services

CDS tool	Difference in practice peformance for preventive service for Black and White patients, coefficient (SE) [95% CI]^a^			
	Aspirin use (n = 187)	Blood pressure control (n = 240)	Cholesterol management (n = 166)	Smoking cessation (n = 179)
Use of registries				
No	1 [Reference]	1 [Reference]	1 [Reference]	1 [Reference]
Yes	−0.93 (0.03) [−6.42 to 4.57]	−0.13 (0.01) [−2.75 to 2.49]	3.11 (0.02) [−1.08 to 7.31]	0.45 (0.02) [−3.32 to 4.22]
Use of preventive guidelines				
Not used or clinician agreement to use	1 [Reference]	1 [Reference]	1 [Reference]	1 [Reference]
Included in EHR prompts or standing orders	−1.75 (0.04) [−9.48 to 5.98]	−3.30 (0.02) [−6.93 to 0.34]	2.92 (0.03) [−2.46 to 8.30]	3.25 (0.02) [−1.76 to 8.27]
Use of chronic guidelines				
Not used or clinician agreement to use	1 [Reference]	1 [Reference]	1 [Reference]	1 [Reference]
Included in EHR prompts or standing orders	−1.05 (0.04) [−8.10 to 6.01]	3.46 (0.02) [−0.16 to 7.09]	−0.04 (0.02) [−4.89 to 4.81]	−3.06 (0.02) [−7.65 to 1.53]

Abbreviations: CDS, clinical decision support; EHR, electronic health record.

^a^ All models adjusted for practice rurality, ownership, and size.

**Table 5.** Multiply Imputed Multivariable Analysis: Association Between CDS Tools and Sex Disparities in Preventive Services^a^

CDS tool	Difference in practice performance for preventive service for men and women, coefficient (SE) [95% CI]			
	Aspirin use (n = 403)	Blood pressure control (n = 450)	Cholesterol management (n = 400)	Smoking cessation (n = 356)
Use of registries				
No	1 [Reference]	1 [Reference]	1 [Reference]	1 [Reference]
Yes	−0.57 (0.01) [−3.38 to 2.23]	−0.66 (0.009) [−2.42 to 1.10]	−0.003 (0.01) [−2.28 to 2.23]	1.13 (0.01) [−1.29 to 3.55]
Use of preventive guidelines				
Not used or clinician agreement to use	1 [Reference]	1 [Reference]	1 [Reference]	1 [Reference]
Included in EHR prompts or standing orders	1.32 (0.02) [−2.97 to 5.61]	−1.69 (0.01) [−4.03 to 0.65]	1.38 (0.02) [−1.61 to 4.37]	−2.67 (0.01) [−5.52 to 0.19]
Use of chronic guidelines				
Not used or clinician agreement to use	1 [Reference]	1 [Reference]	1 [Reference]	1 [Reference]
Included in EHR prompts or standing orders	2.10 (0.02) [−6.10 to 1.90]	0.008 (0.01) [−2.51 to 2.52]	−1.86 (0.01) [−4.83 to 1.11]	3.82 (0.01) [0.95 to 6.68]^b^

Abbreviations: CDS, clinical decision support; EHR, electronic health record.

^a^ All models adjusted for practice rurality, ownership, and size.

^b^ *P* = .009.

### Sensitivity Analysis

The χ^2^ tests used to compare practice characteristics within the analytic sample and excluded practices showed the 2 groups were significantly different in every measure (eTable 1 in Supplement 1). Study practices were more likely to use registries (χ^2^ = 55.2; *P* < .001), to use EHR prompts and standing orders (including CVD prevention guidelines [χ^2^ = 49.2; *P* < .001] and CVD management guidelines [χ^2^ = 45.1; *P* < .001]), and to be clinician owned (χ^2^ = 165.3; *P* < .001) and urban (χ^2^ = 196.3; *P* < .001).

We found disparity estimates among excluded practices were comparable with those of the analytic sample (eTables 5 and 6 in Supplement 1). The 2 exceptions were race disparity estimates for cholesterol management and smoking cessation; however, they were not statistically significant.

When we ran regression models including a category for missing values, we found that missing data were not associated with the disparity estimate in any of the aspirin or blood pressure models (eTable 7 in Supplement 1). They were, however, associated with the disparity estimate in 2 of 3 Black and White models for cholesterol management, and 3 of 3 male and female models for smoking cessation. In total, missing data were associated with the disparity estimate in 5 of 24 models.

Results from regression models with CDS tools coded as 1 variable with 4 levels for practice use of 0, 1, 2, or 3 tools were comparable with Tables 4 and 5 (eTables 8 and 9 in Supplement 1). No clear pattern was observed with an increasing number of CDS tools.

## Discussion

In this cross-sectional study, we set out to evaluate the association between CDS tools and practice-level disparities in primary care CVD preventive services. We found that practices using CDS tools had small disparity estimates that were not statistically significant, though CDS tools were not associated with reductions in race or sex disparities. The exception was practices using CDS tools showed men received higher guideline-concordant care than women in 1 of 3 smoking cessation models. More men smoke compared with women, though men and women are equally likely to receive cessation counseling.^31^

This study adds to the extant literature by using practice-level data to identify structural race and sex disparities in ABCS preventive services among smaller primary care practices. The 576 practices in our study provided ABCS data stratified by sex and/or race. Where race was missing, it was because practices did not provide that to the cooperatives (they could have reported ABCS data by sex). Although we observed substantive missingness in race or sex, we have complete ABCS quality metrics data on hundreds of small primary care clinics, a novel contribution since most primary care studies offer much smaller sample sizes.

We observed FQHCs performed better in providing equitable guideline-concordant care compared with practices that do not focus on the underserved. This may be a function of several factors, including FQHCs’ comprehensive primary care and enabling services, and differences in Centers for Medicare & Medicaid Services reporting requirements and reimbursement structures compared with non-FQHCs. Certain FQHC approaches may warrant wider adoption.

The CDS tools have received little attention for their potential to reduce disparities among smaller primary care practices where most US health care happens. We hypothesized that by identifying due services through automated point-of-care prompts and tracking patient care, CDS tools could provide a mechanism for standardizing patient care and mitigating racial or sex bias. Our hypothesis was not supported, possibly because having registries or prompts may not equate with consistent use, or because even if a practice uses CDS tools, Black patients may have additional access barriers to care.

Including race as a variable in clinical algorithms, such as those incorporated into CDS tools, is subject to debate. Some evidence demonstrates that including race actually exacerbates disparities, others posit it is necessary for optimal clinical decisions.^32,33,34^ A recent study concluded that including a race feature in algorithms for clinical practice would prevent bias when estimating realized outcomes, but increase bias when predicting future outcomes.^35^ We did not have information on algorithms used in practices’ CDS tools in this study; this is an unmeasured factor that may affect findings and should be evaluated in future investigations.

Use of CDS tools is associated with longer appointments and increased cognitive load for clinicians; however, customizing prompts can improve clinician experience.^36,37,38,39^ The quadruple aim^40^ includes clinician well-being, necessitating CDS implementation that supports rather than encumbers clinicians; evidence shows this is possible.^41,42^

We desired to include additional races and ethnicities in our analyses, but were unable due to lack of reporting. Minority group underrepresentation is unfortunately common in clinical studies.^43^ Healthy People 2000, 2010, 2020, and 2030 each aimed to mitigate health disparities—a goal predicated on accurate reporting of race and ethnicity data. Most of this sample participated in stages 1 and 2 of the Medicare Promoting Interoperability Program and used an Office of the National Coordinator for Health Information–certified EHR. Even among such a technologically capable group, over half the practices in EvidenceNOW did not submit data on ABCS quality metrics stratified by race and ethnicity. These practices—using federally certified EHRs—reported inordinate challenges to generating even simple reports for quality improvement, which signals a considerable infrastructure problem.^44,45^ Accordingly, health systems and clinicians could be incentivized to report race and ethnicity data. No such financial incentives are currently offered to US hospitals or clinicians. In the United Kingdom, evidence from a pay-for-performance program demonstrates that incentivizing to increase quality improvement strategies (ie, use of registries) can reduce health inequities, though overall improvements in care were not observed.^46,47,48^

### Limitations

This study has some limitations, including lack of individual patient data, such as comorbidities, patient risk level, and social determinants of health, as they were not feasible within the scope of this initiative. We addressed this by controlling for practice rurality and ownership but are unable to distinguish whether and to what degree differences in individual patient health and social determinants of health contribute to the disparities reported herein. Second, the restriction to practices with sufficient numbers of each patient subgroup being compared substantively reduced the number of practices included in the analyses. Included practices had more Black patients and were more likely to be clinician owned, be located in an urban setting, and use registries, EHR prompts, and standing orders compared with excluded practices. It is possible that because their patient panels are more diverse and they are more likely to use CDS tools, included practices were more predisposed to equitable care delivery, in which case disparities reported herein would be underestimated compared with other practices. We observed relatively high rates of missing race survey data; however, these do not directly influence our regression analyses, which used EHR-derived ABCS quality metrics data stratified by race. Race data on practice patient panels are purely descriptive to characterize the sample; therefore, we do not anticipate response bias. Separately, for stratified ABCS metrics data, missing data were associated with the disparity estimates for 2 of 3 cholesterol models and 3 of 3 smoking cessation models. For these 2 ABCS measures, missing data may distort the disparity estimate. Disparities reported herein, while statistically significant, were small in absolute terms. However, adult CVD prevalence is nearly 50%, with Black patients disproportionately affected, so addressing any disparity in guideline-concordant care could achieve gains in health equity. Third, we know that ownership makes a difference in the operations and potentially outcomes of a practice. Limited sample size affected our ability to understand whether ownership is an effect modifier between disparities and CDS tools, though we strongly hypothesize that it is. Fourth, these results are subject to possible omitted variable bias, although we included covariates previously found to affect care equity. Additionally, we were unable to characterize how practices use CDS tools, as this variable was dichotomous; future research is needed in this area. Furthermore, as a cross-sectional study, causation cannot be determined from these results.

## Conclusion

To our knowledge, this cross-sectional study is the first to demonstrate structural race and sex disparities in ABCS preventive services in a large primary care setting. These findings may be of interest to clinicians, clinical leadership, and policy makers evaluating health information technology-based options for reducing health care disparities.

## Notes

Supplement 1. **eFigure.** EvidenceNOW Cohort **eTable 1.** Sensitivity Analysis—Comparative Analysis of Practices Included and Excluded From Analytic Sample **eTable 2.** Study Outcome Measures and ABCS Clinical Guideline Definitions From Centers for Medicare & Medicaid Services **eTable 3.** Sample Health Disparities—Stratified Analyses by FQHC Status by Race (N = 576) **eTable 4.** Sample Health Disparities—Stratified Analyses by FQHC Status by Sex (N = 576) **eTable 5.** Sensitivity Analysis—Disparities in Guideline-Concordant Care by Race for Practices That Did Not Submit Survey Data (n = 207) **eTable 6.** Sensitivity Analysis—Disparities in Guideline-Concordant Care by Sex for Practices That Did Not Submit Survey Data (n = 207) **eTable 7.** Sensitivity Analysis—Linear Regression Modeling the Relationship Between Health Disparities and Clinical Decision Support Tools, Including Missing Data Category (N = 576) **eTable 8.** Sensitivity Analysis—Multiply-Imputed Multivariable Analysis: Association Between Number of Clinical Decision Support Tools and Racial Disparities in ABCS Preventive Services (N = 576) **eTable 9.** Sensitivity Analysis—Multiply-Imputed Multivariable Analysis: Association Between Number of Clinical Decision Support Tools and Sex Disparities in ABCS Preventive Services (N = 576)

Supplement 2.
Data Sharing Statement
