# Supplementary material for: Differences in Use of Clinical Decision Support Tools and Implementation of Aspirin, Blood Pressure Control, Cholesterol Management, and Smoking Cessation Quality Metrics in Small Practices by Race and Sex
Source: JAMA Netw Open. 2023 Aug 2;6(8):e2326905. doi: 10.1001/jamanetworkopen.2023.26905 (PMC10398408; doi:10.1001/jamanetworkopen.2023.26905)
Supplement: Supplement 1. — eFigure. EvidenceNOW Cohort eTable 1. Sensitivity Analysis—Comparative Analysis of Practices Included and Excluded From Analytic Sample eTable 2. Study Outcome Measures and ABCS Clinical Guideline Definitions From Centers for Medicare & Medicaid Services eTable 3. Sample Health Disparities—Stratified Analyses by FQHC Status by Race (N = 576) eTable 4. Sample Health Disparities—Stratified Analyses by FQHC Status by Sex (N = 576) eTable 5. Sensitivity Analysis—Disparities in Guideline-Concordant Care by Race for Practices That Did Not Submit Survey Data (n = 207) eTable 6. Sensitivity Analysis—Disparities in Guideline-Concordant Care by Sex for Practices That Did Not Submit Survey Data (n = 207) eTable 7. Sensitivity Analysis—Linear Regression Modeling the Relationship Between Health Disparities and Clinical Decision Support Tools, Including Missing Data Category (N = 576) eTable 8. Sensitivity Analysis—Multiply-Imputed Multivariable Analysis: Association Between Number of Clinical Decision Support Tools and Racial Disparities in ABCS Preventive Services (N = 576) eTable 9. Sensitivity Analysis—Multiply-Imputed Multivariable Analysis: Association Between Number of Clinical Decision Support Tools and Sex Disparities in ABCS Preventive Services (N = 576) [file jamanetwopen-e2326905-s001.pdf]

Supplementary Online Content

Roberts MM, Marino M, Wells R, Atem FD, Balasubramanian BA. Differences in use of clinical decision support tools and implementation of aspirin, blood pressure control, cholesterol management, and smoking cessation quality metrics in small practices by race and sex. *JAMA Netw Open*. 2023;6(8):e2326905. doi:10.1001/jamanetworkopen.2023.26905

eFigure. EvidenceNOW Cohort
eTable 1. Sensitivity Analysis—Comparative Analysis of Practices Included and Excluded From Analytic Sample
eTable 2. Study Outcome Measures and ABCS Clinical Guideline Definitions From Centers for Medicare & Medicaid Services
eTable 3. Sample Health Disparities—Stratified Analyses by FQHC Status by Race (N = 576)
eTable 4. Sample Health Disparities—Stratified Analyses by FQHC Status by Sex (N = 576)
eTable 5. Sensitivity Analysis—Disparities in Guideline-Concordant Care by Race for Practices That Did Not Submit Survey Data (n = 207)
eTable 6. Sensitivity Analysis—Disparities in Guideline-Concordant Care by Sex for Practices That Did Not Submit Survey Data (n = 207)
eTable 7. Sensitivity Analysis—Linear Regression Modeling the Relationship Between Health Disparities and Clinical Decision Support Tools, Including Missing Data Category (N = 576)
eTable 8. Sensitivity Analysis—Multiply-Imputed Multivariable Analysis: Association Between Number of Clinical Decision Support Tools and Racial Disparities in ABCS Preventive Services (N = 576)
eTable 9. Sensitivity Analysis—Multiply-Imputed Multivariable Analysis: Association Between Number of Clinical Decision Support Tools and Sex Disparities in ABCS Preventive Services (N = 576)

This supplementary material has been provided by the authors to give readers additional information about their work.

© 2023 Roberts MM et al. *JAMA Network Open*.

eFigure. EvidenceNOW Cohort

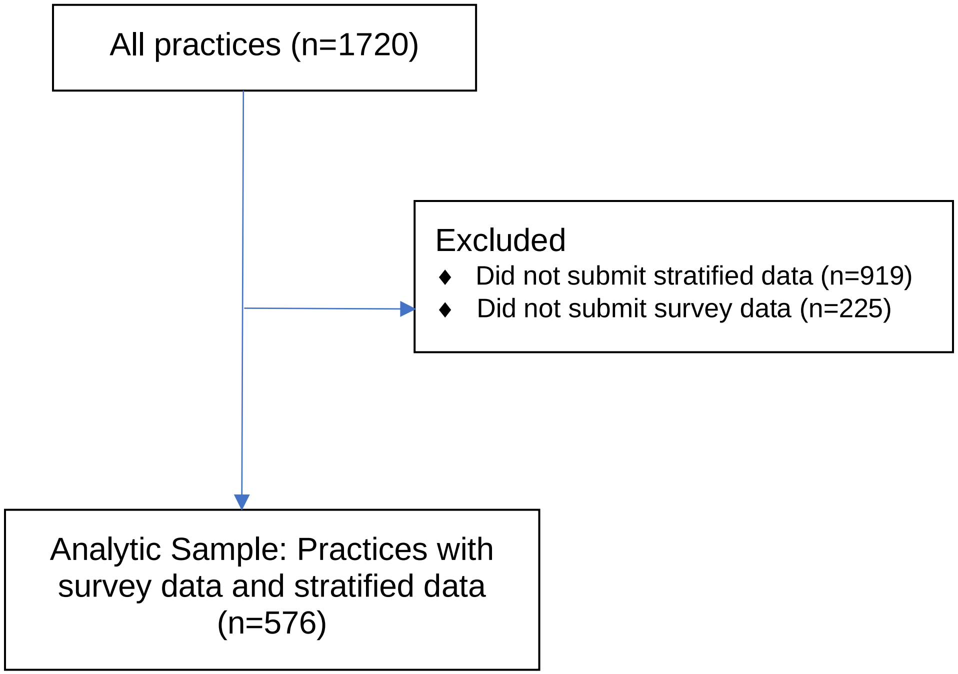

© 2023 Roberts MM et al. *JAMA Network Open*.

eTable 1. Sensitivity Analysis—Comparative Analysis of Practices Included and Excluded From Analytic Sample

Practice Characteristic	Excluded (Did not submit ABCS stratified data) (n=919 practices)	Analytic Sample (Submitted both stratified data and survey data) (n=576 practices)	Chi-Square Statistic	p-value
Practice location			196.3	<0.001
Urban	527 (46.1)	424 (73.6)		
Suburban	58 (5.1)	49 (8.5)		
Large Town	164 (14.3)	38 (6.6)		
Rural Area	170 (14.9)	65 (11.3)		
Practice size, n (%)			33.2	<0.001
Solo practice	207 (18.1)	150 (26.0)		
2-5 clinicians	476 (41.6)	223 (38.7)		
6-10 clinicians	134 (11.7)	71 (12.3)		
11 or more clinicians	94 (8.2)	65 (11.3)		
Practice ownership, n (%)			165.3	<0.001
Clinician owned	363 (31.7)	290 (50.4)		
Hospital/Health-System-Owned/HMO	246 (21.5)	116 (20.1)		
FHQC	207 (18.1)	60 (10.4)		
Other	26 (2.3)	62 (10.8)		
Classified as a Medically Underserved Area, n (%)			33.0	<0.001
Yes	360 (31.5)	240 (41.7)		
No	503 (44.0)	257 (44.6)		
Received external incentives in prior 12 months			130.3	<0.001
Yes	416 (36.4)	328 (56.9)		
No	228 (19.3)	155 (26.9)		
Participation in other demonstration projects			9.09	0.01
Yes	279 (24.4)	163 (28.3)		
No	636 (55.6)	276 (47.9)		

© 2023 Roberts MM et al. *JAMA Network Open*.

ONC-certified EHR			11.7	0.003
Yes	778 (68.01)	437 (75.9)		
No	11 (1.0)	3 (0.5)		
Participation in Meaningful Use			14.6	0.002
Not participating	173 (15.1)	58 (10.1)		
Stage 1 only	101 (8.8)	76 (13.2)		
Stage 1 and Stage 2	583 (51.0)	302 (52.4)		
Produced quality report(s) in prior 6 months			119.2	<0.001
Yes	494 (43.2)	402 (69.8)		
No	187 (16.4)	76 (13.2)		
Use of at least one registry			55.2	<0.001
Yes	553 (48.3)	380 (66.0)		
No	363 (31.7)	97 (16.8)		
Guidelines for CVD prevention			49.2	<0.001
Not used or clinician agreement to use	399 (34.9)	122 (21.2)		
Included in EHR prompts or standing orders	518 (45.3)	361 (62.7)		
Guidelines for CVD management			45.1	<0.001
Not used or clinician agreement to use	436 (38.1)	144 (25.0)		
Included in EHR prompts or standing orders	481 (42.1)	339 (58.9)		

*Percentages may not sum to 100% due to missing data

© 2023 Roberts MM et al. *JAMA Network Open*.

eTable 2. Study Outcome Measures and ABCS Clinical Guideline Definitions From Centers for Medicare & Medicaid Services

Outcome Measure	Numerator	Denominator
Aspirin	Patients meeting aspirin denominator criteria who have documentation of use of aspirin or another antithrombotic during the measurement period. (CMS164v4)	CMS164v4 - Patients 18 years of age and older with a visit during the measurement period, and an active diagnosis of ischemic vascular disease (IVD) or who were discharged alive for acute myocardial infarction (AMI), coronary artery bypass graft (CABG) or percutaneous coronary interventions (PCI) in the 12 months prior to the measurement period.
Blood Pressure Control	Patients meeting blood pressure denominator criteria whose blood pressure at the most recent visit is adequately controlled (systolic blood pressure < 140 mmHg and diastolic blood pressure < 90 mmHg) during the measurement period. (CMS165v4)	CMS165v4 - Patients 18-85 years of age who had a diagnosis of essential hypertension within the first six months of the measurement period or any time prior to the measurement period.
Cholesterol Management	Composite statin therapy measure that includes patients meeting cholesterol management denominator criteria who were prescribed or were on statin therapy during the measurement period. (CMS 347)	Composite statin therapy measure that includes patients considered at high risk of cardiovascular events defined as:<br>patients ages >= 21 years at the beginning of the measurement period with clinical ASCVD diagnosis;<br>OR patients ages >= 21 years at the beginning of the measurement period who have ever had a fasting or direct

© 2023 Roberts MM et al. *JAMA Network Open*.

		laboratory result of LDL-C >= 190 mg/dL;<br>OR patients ages 40 to 75 years at the beginning of the measurement period with Type 1 or Type 2 diabetes and with a LDL-C result of 70–189 mg/dL recorded as the highest fasting or direct laboratory test result in the measurement year or during the two years prior to the beginning of the measurement period)
Smoking Cessation Support	Patients who were screened for tobacco use at least once within 24 months AND who received tobacco cessation intervention if identified as a tobacco user. (CMS138v4)	Patients aged 18 years and older seen for at least two visits or at least one preventive visit during the measurement period.

© 2023 Roberts MM et al. *JAMA Network Open*.

eTable 3. Sample Health Disparities—Stratified Analyses by FQHC Status by Race (N = 576)

FQHC Practices, n=60						Non-FQHC Practices, n=516					
	White Mean % (SD)	Black Mean % (SD)	Disparity Estimate Mean % (SD)	95% CI	*t*-statistic		White Mean % (SD)	Black Mean % (SD)	Disparity Estimate Mean % (SD)	95% CI	*t*-statistic
**Aspirin,** n=29 practices	48.10 (0.31)	51.86 (0.26)	-3.76 (0.18)	(-10.61, 3.09)	-1.12	**Aspirin,** n=158 practices	61.98 (0.01)	61.98 (0.29)	0.006 (0.11)	(-1.66, 1.67)	0.007
**Blood Pressure Control,** n=40 practices	77.11 (0.18)	71.0 (0.21)	6.12 (0.06)	(4.09, 8.14)	6.11***	**Blood Pressure Control,** n=200 practices	66.27 (0.12)	61.31 (0.13)	4.96 (0.07)	(4.01, 5.92)	10.23***
**Cholesterol Management,** n=28 practices	55.25 (0.26)	55.34 (0.22)	-0.09 (0.10)	(-3.95, 3.78)	-0.05	**Cholesterol Management, ,** n=138 practices	63.59 (0.18)	61.78 (0.17)	1.81 (0.09)	(0.23, 3.39)	2.27*
**Smoking Cessation,** n=46 practices	51.12 (0.33)	53.12 (0.35)	-1.99 (0.06)	(-3.69, -0.30)	-2.37*	**Smoking Cessation,** n=133 practices	55.43 (0.35)	55.96 (0.37)	-0.53 (0.07)	(-1.66, 0.59)	-0.94

*p<0.05 **p<0.01 ***p<0.001

© 2023 Roberts MM et al. *JAMA Network Open*.

eTable 4. Sample Health Disparities—Stratified Analyses by FQHC Status by Sex (N = 576)

**FQHC Practices, n=60**						**Non-FQHC Practices, n=516**					
	Male Mean % (SD)	Female Mean % (SD)	Disparity Estimate Mean % (SD)	95% CI	*t*-statistic		Male Mean % (SD)	Female Mean % (SD)	Disparity Estimate Mean % (SD)	95% CI	*t*-statistic
**Aspirin,** n=43 practices	50.69 (0.29)	52.95 (0.29)	-2.26 (0.13)	(-6.19, 1.67)	-1.16	**Aspirin,** n=360 practices	61.50 (0.29)	56.34 (0.28)	5.15 (0.10)	(4.14, 6.17)	9.98***
**Blood Pressure Control,** n=53 practices	70.56 (0.17)	72.75 (0.17)	-2.19 (0.04)	(-3.38, -1.01)	-3.70***	**Blood Pressure Control,** n=397 practices	64.68 (0.13)	66.42 (0.13)	-1.74 (0.06)	(-2.32, -1.17)	-6.01***
**Cholesterol Management,** n=45 practices	56.11 (0.20)	56.13 (0.21)	-0.02 (0.06)	(-1.92, 1.89)	-0.02	**Cholesterol Management,** n=355 practices	62.06 (0.20)	57.68 (0.19)	4.38 (0.08)	(3.59, 5.17)	10.88***
**Smoking Cessation,** n=52 practices	48.59 (0.35)	49.14 (0.33)	-0.55 (0.07)	(-2.61, 1.50)	-0.54	**Smoking Cessation,** n=303 practices	49.63 (0.33)	51.49 (0.33)	-1.86 (0.07)	(-2.62, -1.10)	-4.80***

*p<0.05 **p<0.01 ***p<0.001

© 2023 Roberts MM et al. *JAMA Network Open*.

eTable 5. Sensitivity Analysis—Disparities in Guideline-Concordant Care by Race for Practices That Did Not Submit Survey Data (n = 207)

	White Mean (SE)	Black Mean (SE)	Disparity Estimate Mean (SE)	95% CI	*t*-statistic
Aspirin, n=29 practices	50.68 (0.05)	48.16 (0.06)	2.52 (0.02)	(-1.65, 6.69)	1.24
Blood Pressure Control, n=61 practices	68.27 (0.01)	61.81 (0.02)	6.46 (0.007)	(4.96, 7.96)	8.63***
Cholesterol Management, n=37 practices	52.78 (0.03)	54.40 (0.03)	-1.62 (0.03)	(-6.95, 3.70)	-0.62
Smoking Cessation, n=17 practices	46.00 (0.08)	44.86 (0.08)	1.15 (0.02)	(-3.89, 6.19)	0.48

*p<0.05 **p<0.01 ***p<0.001

eTable 6. Sensitivity Analysis—Disparities in Guideline-Concordant Care by Sex for Practices That Did Not Submit Survey Data (n = 207)

	Male Mean (SE)	Female Mean (SE)	Disparity Estimate Mean (SE)	95% CI	*t*-statistic
Aspirin, n=115 practices	58.53 (0.03)	54.47 (0.03)	4.06 (0.01)	(2.12, 5.99)	4.15***
Blood Pressure Control, n=134 practices	65.84 (0.01)	68.50 (0.01)	-2.66 (0.005)	(-3.62, -1.69)	-5.44***
Cholesterol Management, n=145 practices	57.92 (0.02)	55.52 (0.02)	2.40 (0.01)	(0.16, 4.64)	2.12*
Smoking Cessation, n=85 practices	36.69 (0.03)	40.52 (0.03)	-3.84 (0.008)	(-5.47, -2.20)	-4.66***

*p<0.05 **p<0.01 ***p<0.001

© 2023 Roberts MM et al. *JAMA Network Open*.

eTable 7. Sensitivity Analysis—Linear Regression Modeling the Relationship Between Health Disparities and Clinical Decision Support Tools, Including Missing Data Category (N = 576)

White/Black Difference	Aspirin, n=187		Blood Pressure, n=240		Cholesterol, n=166		Smoking, n=179	
	Coefficient (SE)	95% CI	Coefficient (SE)	95% CI	Coefficient (SE)	95% CI	Coefficient (SE)	95% CI
**Use of registries**								
No	ref	ref	ref	ref	ref	ref	ref	ref
Yes	-1.16 (0.02)	(-5.88, 3.56)	-0.02 (0.01)	(-2.37, 2.32)	4.26 (0.02)	(0.24, 8.27)	-0.01 (0.01)	(-2.92, 2.90)
Missing	-0.91 (0.03)	(-7.09, 5.26)	-0.20 (0.02)	(-3.24, 2.84)	5.55 (0.03)	(0.31, 10.79)	-1.77 (0.02)	(-5.39, 1.85)
**Use of preventive guidelines**								
Not used or clinician agreement to use	ref	ref	ref	ref	ref	ref	ref	ref
Included in EHR prompts or standing orders	-2.96 (0.02)	(-7.26, 1.33)	-0.12 (0.01)	(-2.30, 2.05)	3.37 (0.02)	(-0.30, 7.05)	-0.20 (0.01)	(-2.60, 2.19)
Missing	-1.07 (0.03)	(-7.10, 4.95)	-1.92 (0.01)	(-4.86, 1.03)	5.21 (0.03)	(0.11, 10.32)	-2.47 (0.02)	(-5.67, 0.73)
**Use of chronic guidelines**								
Not used or clinician agreement to use	ref	ref	ref	ref	ref	ref	ref	ref
Included in EHR prompts or standing orders	-2.86 (0.02)	(-6.86, 1.13)	0.98 (0.01)	(-1.04, 3.01)	2.61 (0.02)	(-0.80, 6.03)	-1.00 (0.01)	(-3.19, 1.27)
Missing	-0.80 (0.03)	(-6.58, 4.97)	-1.14 (0.01)	(-3.95, 1.68)	4.46 (0.02)	(-0.43, 9.34)	2.99 (0.02)	(-6.05, 0.06)

*highlighted results indicate missingness significantly contributed to the model

Male/Female Difference	Aspirin, n=403		Blood Pressure, n=450		Cholesterol, n=400		Smoking, n=356	
	Coefficient (SE)	95% CI	Coefficient (SE)	95% CI	Coefficient (SE)	95% CI	Coefficient (SE)	95% CI
**Use of registries**								
No	ref	ref	ref	ref	ref	ref	ref	ref
Yes	-0.59 (0.01)	(-3.40, 2.23)	-0.82 (0.007)	(-2.26, 0.63)	0.04 (0.01)	(-1.99, 2.08)	1.35 (0.01)	(-0.73, 3.42)
Missing	0.51 (0.02)	(-3.00, 4.02)	0.14 (0.009)	(-1.63, 1.91)	-0.74 (0.01)	(-3.23, 1.75)	2.92 (0.01)	(0.36, 5.47)
**Use of preventive guidelines**								
Not used or clinician agreement to use	ref	ref	ref	ref	ref	ref	ref	ref
Included in EHR prompts or standing orders	-0.41 (0.01)	(-2.93, 2.12)	-1.12 (0.007)	(-2.44, 0.20)	0.21 (0.01)	(-1.69, 2.11)	0.92 (0.009)	(-0.88, 2.72)
Missing	0.87 (0.02)	(-2.52, 4.25)	-0.23 (0.009)	(-1.91, 1.46)	-1.25 (0.01)	(-3.66, 1.17)	2.97 (0.01)	(0.61, 5.33)
**Use of chronic guidelines**								
Not used or clinician agreement to use	ref	ref	ref	ref	ref	ref	ref	ref
Included in EHR prompts or standing orders	-0.71 (0.01)	(-3.09, 1.68)	-0.70 (0.006)	(-1.96, 0.55)	-0.30 (0.009)	(-2.08, 1.49)	2.04 (0.009)	(0.35, 3.73)
Missing	0.68 (0.01)	(-2.58, 3.95)	0.12 (0.008)	(-1.51, 1.75)	-1.61 (0.01)	(-3.92, 0.70)	3.72 (0.01)	(1.46, 5.98)

© 2023 Roberts MM et al. *JAMA Network Open*.

*highlighted results indicate missingness significantly contributed to the model

© 2023 Roberts MM et al. *JAMA Network Open*.

eTable 8. Sensitivity Analysis—Multiply-Imputed Multivariable Analysis: Association Between Number of Clinical Decision Support Tools and Racial Disparities in ABCS Preventive Services (N = 576)

Number of CDS Tools Used by a Practice	Aspirin, n=187 Coefficient (SE); 95% CI	Blood Pressure Control, n=240 Coefficient (SE); 95% CI	Cholesterol Management, n=166 Coefficient (SE); 95% CI	Smoking Cessation, n=179 Coefficient, (SE); 95% CI
0	Ref	ref	ref	ref
1	3.31 (0.04); (-4.86, 11.48)	-0.31 (0.02); (-4.59, 3.97)	-1.50 (0.03); (-8.34, 5.34)	1.08 (0.03); (-4.59, 6.75)
2	-0.49 (0.04); (-8.72, 7.75)	-3.34 (0.02); (-7.59, 0.90)	1.77 (0.03); (-4.89, 8.44)	1.42 (0.03); (-4.27, 7.12)
3	-0.86 (0.04); (-8.04, 6.33)	-0.69 (0.02); (-4.03, 2.64)	3.71 (0.03); (-2.11, 9.54)	0.34 (0.03); (-4.73, 5.40)

eTable 9. Sensitivity Analysis—Multiply-Imputed Multivariable Analysis: Association Between Number of Clinical Decision Support Tools and Sex Disparities in ABCS Preventive Services (N = 576)

Number of CDS Tools Used by a Practice	Aspirin, n=403 Coefficient (SE); 95% CI	Blood Pressure Control, n=450 Coefficient (SE); 95% CI	Cholesterol Management, n= 400 Coefficient (SE); 95% CI	Smoking Cessation, n=356 Coefficient (SE); 95% CI
0	ref	ref	ref	ref
1	1.96 (0.02); (-2.44, 6.36)	-0.09 (0.01); (-2.85, 2.68)	0.37 (0.02); (-2.98, 3.72)	3.00 (0.02); (-0.42, 6.42)
2	2.09 (0.02); (-2.50, 6.68)	-0.37 (0.02); (-3.47, 2.72)	0.90 (0.02); (-2.50, 4.30)	2.02 (0.02); (-1.53, 5.58)
3	0.83 (0.02); (-3.16, 4.83)	-1.16 (0.01); (-3.46, 1.13)	0.11 (0.02); (-2.92, 3.13)	3.56 (0.02); (0.55, 6.57)

All models adjusted for practice rurality, ownership, and size
Highlighting indicates statistically significant result

© 2023 Roberts MM et al. *JAMA Network Open*.
