# Supplementary material for: Differences in Use of Clinical Decision Support Tools and Implementation of Aspirin, Blood Pressure Control, Cholesterol Management, and Smoking Cessation Quality Metrics in Small Practices by Race and Sex
Source: JAMA Netw Open. 2023 Aug 2;6(8):e2326905. doi: 10.1001/jamanetworkopen.2023.26905 (PMC10398408; doi:10.1001/jamanetworkopen.2023.26905)
Supplement: Supplement 2. — Data Sharing Statement [file jamanetwopen-e2326905-s002.pdf]

Data Sharing Statement

Roberts. Differences in Use of Clinical Decision Support Tools and Implementation of Aspirin, Blood Pressure Control, Cholesterol Management, and Smoking Cessation Quality Metrics in Small Practices by Race and Sex. *JAMA Netw Open*. Published August 02, 2023. doi:10.1001/jamanetworkopen.2023.26905

Data
Data available: No
